# Living well with dementia: a qualitative interview study on family caregivers’ call for more person- and family-centered dementia support

**DOI:** 10.1186/s12877-025-06429-z

**Published:** 2025-10-06

**Authors:** Pia Bastholm-Rahmner, Katharina Schmidt-Mende, Karin Modig, Monica Bergqvist

**Affiliations:** 1 Academic Primary Health Care Centre, Region Stockholm, Sweden; 2 Department of Laboratory Medicine, Division of Clinical Pharmacology, Karolinska Institutet, Stockholm, Sweden; 3 Department of Neurobiology and Care Sciences and Society, Division of Family Medicine and Primary Care , Karolinska Institutet, Stockholm, Sweden; 4 Institute of Environmental Medicine, Unit of Epidemiology, Karolinska Institutet, Stockholm, Sweden; 5 Department of Neurobiology, Care Sciences and Society, Division of Nursing , Karolinska Institutet, Stockholm, Sweden

**Keywords:** Coping, Dementia care, Family caregivers, Everyday life, Person centered care, Qualitative research

## Abstract

**Background:**

Family members are often the primary caregivers for individuals with dementia, but they face significant challenges in navigating health care and social services, especially as the disease progresses. Many caregivers experience loneliness, social isolation, and stress from sacrificing their own well-being. This study aimed to describe the daily experiences of family caregivers of individuals with dementia, with the objective of better understanding and addressing their specific needs.

**Methods:**

Qualitative interviews with 11 family caregivers in Sweden. Data were analyzed by inductive thematic analysis.

**Results:**

Three themes were identified: (1) *Struggling with conflicting emotions and social challenges -* caregivers reported experiencing stress, physical exhaustion, and emotional strain due to constant availability and the challenges of managing behavioral changes, further intensified by isolation and shrinking social networks. (2) *Balancing autonomy in care decisions -* caregivers described the paradox of bearing full responsibility for care decisions despite having limited access to information, and, (3) *Dependence on home care and nursing homes that are not adapted to needs* - caregivers expressed a reliance on home care and nursing homes, yet noted that these services are often ill-equipped to address the specific demands of dementia care.

**Conclusion:**

Caring for a family member with dementia reshapes family roles and creates ongoing challenges in balancing safety, well-being, and limited support. This study highlights the need for person- and family-centered care, grounded in the home context and developed in partnership with family caregivers. Improved coordination, trained staff, and supportive policies are essential, alongside continued conceptual development to define best practices in dementia care.

## Background

Globally, more than 55 million people are currently living with dementia, affecting 8.1% of women and 5.4% of men over 65 years [1]. In Sweden, approximately 150,000 are living with dementia [2–4]. For most of these individuals, family members serve as the primary caregivers, providing support at home [2]. As the disease progresses, it leads to a gradual decline in cognitive, behavioral, and psychological functioning. While most people with dementia prefer to remain in their own homes, support from family caregivers becomes necessary to enable this [2, 5, 6].

Caring for a family member with dementia is a journey filled with challenges. Family caregivers often find themselves struggling to navigate a complex maze of health care providers and social services, particularly during critical stages such as recognizing the first symptoms, receiving a dementia diagnosis and in the progression of the disease [7–10]. In this process, both the family caregivers and the person with dementia have a significant need for support in finding the right care options and navigating the system. Without this assistance, they may feel disoriented and uninformed about the available resources [6, 11]. Key factors contributing to family caregivers burden include the severity of the dementia, behavioral disturbances, changes in personality, and the presence of psychiatric symptoms in the person with dementia [12]. Despite this, caregiving is perceived positively if there is formal support from health care professionals combined with a strong network of family and friends [11]. Many family caregivers experience high levels of loneliness and social isolation [13, 14] as they frequently feel compelled to sacrifice their own interests, relationships, and well-being in the course of caregiving [10, 11]. There may be differences in how spouse and non-spouse caregivers perceive what support they need from health care, friends and family [15, 16]. Spouse caregivers often provide more intensive care, with less support, and face greater psychological, physical, and social burdens than non-spouse caregivers [15]. While previous research has outlined the types of support needed by family caregivers, such as competent home care, day care services and support groups with other family caregivers [11, 17], there remains a gap in understanding their daily lives and how they experience care and support when caring for a family member with dementia. Delving into these lived experiences can offer valuable insights into the practical and emotional challenges they face and ultimately providing suggestions for improvements.

To mitigate the negative consequences associated with caring for a person with dementia, support for family caregivers is essential. However, research shows that the support provided is often not tailored to the family caregivers’ actual needs [18]. While previous studies have largely focused on identifying what types of support should be offered such as home care, day care services, or support groups there is a lack of in-depth knowledge about how family caregivers themselves experience their everyday lives and the support they receive or seek. Few studies have explored family caregivers’ personal narratives about their daily realities, emotional strain, and social challenges in relation to the support systems available to them. To better respond to the needs of family caregivers, further research is needed that draws on their lived experiences and captures the complexity of caring, coping, and navigating support in everyday life. Therefore, this study aims to describe the everyday lives and experiences of family caregivers, to better understand and address their needs when caring for a family member with dementia.

## Method

### Study design

The study was designed using a qualitative approach which is well suited for exploring family caregivers lived experiences. Data collection was carried out through semi-structured individual interviews [19], and an inductive thematic approach was utilized for the analysis [20]. The analysis was conducted using a constructivist framework, which recognizes that knowledge is formed through personal lived experiences. It also acknowledges that researchers’ own experiences can shape the knowledge generated through interactions with participants and data [21]. This approach requires researchers to accept the informants’ narratives without imposing preconceived ideas or making comparisons to established facts. This study’s data was collected as part of a broader project, in which some interviews were conducted with both the person with dementia and a family caregiver, while others were conducted with only the family caregiver. Although the original project aimed to explore ways to improve primary health care, many family caregivers naturally turned the conversation to their daily experiences and challenges together with the person with dementia. When these experiences were shared, the interviewer asked follow-up questions, and these reflections form the core findings of this study. The Regional Ethical Review Board in Sweden, (Reg. no. 2023-05475-01) approved the study.

### Reference group

The research team collaborates with a reference group from the Swedish Dementia Association (www.demensforbundet.se), which is politically and religiously independent. The organization works to improve conditions for persons with dementia and their relatives. The reference group includes both staff working for the association, persons living with dementia, and relatives, who have contributed their perspectives throughout the study. Their involvement included assisting with the recruitment of informants and discussion of interview questions. Although they did not participate in the data analysis, they provided valuable feedback on the findings. We shared a preliminary version of the findings with the Swedish Dementia Association after the analysis was completed in order to gather feedback on the relevance and clarity of the results from a user perspective.

### Recruitment and participants

Family caregivers were either adult children or spouses of persons diagnosed with dementia. All informants were recruited in collaboration with the Swedish Dementia Association Stockholm. The recruitment process involved the organization disseminating information about the research project through its channels, both digital and in-person. Interested individuals were invited to contact the research team for more details. Thirteen persons from Region Stockholm were willing to participate in the study. Interviews were conducted with 11 family caregivers (see Table 1). Of the two interested persons who did not participate, one could not be reached for further planning, and the other was hospitalized.

**Table 1:** Demographic characteristics of informants (*n* = 11)

Characteristic	
Gender	Male: 4 Female: 7
Age (years)	Mean: 75 Range: 55–87
Education Level	Pre-secondary/Secondary: 3 University: 8
Country of Birth	Sweden: 9 Abroad: 2
Spouse	9
Adult child	2

### Data collection

Between February and April 2024, two of the authors (MB and PBR) conducted semi-structured interviews [19] with family caregivers. All interviews were conducted in Swedish and took place in the informants’ homes, at locations in the Stockholm Region, or at the offices of the Swedish Dementia Association.

Before the interview, informants interested in taking part received both oral and written information about the study’s aim and the intended use of its results, along with an opportunity to ask questions. They were also informed that they could withdraw from the interview at any point and that their participation would remain confidential, with all names and locations removed from the transcripts. Each informant provided their consent by signing an informed consent form.

To initiate and structure the interviews an interview guide with predefined topics was generated from previous research and in discussions with the reference group. To cover different aspects of experiences, the interview started with broad open-ended questions (Table 2). Data collection continued until information power was reached [22], i.e. when sufficient data had been collected to develop a robust and valid understanding of the aim. Using the concept of information power, we determined our aim to be narrow, and that dense sample specificity could be achieved by recruiting informants with substantial experience in the issue, supporting a lower sample size. Therefore, we considered 11 respondents to be adequate to address our research objectives.

**Table 2:** Interview guide

1. Can you tell me about your caregiving role?
2. What are the most challenging aspects of daily caregiving?
3. What kind of external support do you receive?
4. What kind of additional support or resources would make your caregiving role easier?

Each interview was recorded and transcribed verbatim, with sessions lasting between 56 and 92 min, averaging 71 min.

### Data analysis

The transcribed interviews were analyzed using a thematic analysis, a method for identifying, examining, and conveying patterns of significance within the data [20]. Given the manageable size of the dataset, the analysis was conducted manually to allow for close engagement with the data; qualitative software was therefore not deemed necessary. First, transcripts were read several times to grasp the entire material. All data relevant to the study’s aim, concerning the informants’ everyday experiences, was organized through systematic coding of data extracts that reflected meaningful patterns in relation to the research focus. Initially, two qualitative researchers (MB and PBR) separately coded and collated codes with similar content into potential subthemes. These authors discussed how potential subthemes were related by similar content or processes, and generated potential themes, see Table 3 for an example of the analytic process.

**Table 3:** Example of the analytical process for the theme “struggling with conflicting emotions and social challenges”

Quote	Code	Subtheme	Theme
“I’m always on alert. Even during the night, I can’t relax because I have to be available if something happens.”	Being constantly available is exhausting	Constant availability creates stress and physical exhaustion	Struggling with conflicting emotions and social challenges
“Sometimes I get angry, and then I feel guilty… I know it’s not his fault.”	Ambivalence and guilt in emotional response	Mixed feelings in coping with behavioral changes	
“Friends have stopped calling. They don’t understand what it’s like, and I don’t have time anyway.”	Social withdrawal and lack of support	Isolation and shrinking social networks due to societal stigma and sole caregiving	

When opinions differed, parts of the transcripts were reread. The researchers reviewed potential subthemes during repeated discussions and merged subthemes considered to have a common origin, constructed refined themes, and identified illustrative quotes in the data. The other researchers (KM and KSM) conducted reciprocal reading between transcripts and themes/subthemes to ensure no data was overlooked, to further refine the name and definition of each theme. Quotes were translated from Swedish to English by one of the co-authors (KM).

To ensure credibility, the research team adhered to Braun and Clarke’s guidelines [20] and followed the consolidated criteria for reporting qualitative research (COREQ) [23]. Throughout the analytical process, discussions involving all authors were held to reinforce the trustworthiness of the findings.

## Results

Analysis identified three themes, each with subthemes, who capture family caregivers’ daily experiences (Table 4).

**Table 4:** Themes and sub-themes of the daily experiences of family caregivers providing care for a person with dementia

Theme	Subtheme
Struggling with conflicting emotions and social challenges	- Constant availability creates stress and physical exhaustion - Mixed feelings in coping with behavioral changes - Isolation and shrinking social networks due to societal stigma and sole caregiving
Balancing autonomy in care decisions	- Paradox of full responsibility with limited information - Challenges for the person with dementia in recognizing their own care needs - Preference to entrust care decisions to professionals
Dependence on home care and nursing homes that are not adapted to needs	- Need for home care with dementia expertise, continuity and familiarity - High threshold for qualifying for nursing home placement

### Struggling with conflicting emotions and social challenges

#### Constant availability creates stress and physical exhaustion

Family caregivers believe that a person with dementia has a better quality of life in a safe, familiar home environment than in a nursing home but caring for a family member with dementia is both emotionally and physically exhausting. People in their social circles often advise them to avoid overexerting themselves and to seek for assistance from health care providers or local authorities, such as home care or nursing home. Despite this, many informants chose to decline help, feeling a sense of obligation to manage the caregiving responsibilities on their own. As a result, they continuously push their physical and emotional limits, often taking on physically demanding tasks, such as assisting the person with daily activities and personal hygiene.

*Female family caregiver: I kept pushing my limits all the time. When it was at its worst*,* he would only sleep for two hours at night. And so did I*,* because he would get up and move furniture. But I declined home care because I thought I should manage it on my own.*

The family caregivers feel a responsibility for constantly being available and to meet the needs of the person with dementia, and the responsibility for safety rests with them. They cannot afford to get sick or be absent because the person with dementia may become anxious, injured, or overdose medication. Family caregivers describe how they adjust their daily lives in parallel with the progression of the disease and at the same time emphasize the importance of maintaining routines.

#### Mixed feelings in coping with behavioral changes

Persons with dementia can exhibit behavioral changes including mood swings, increased anger and suspicion, and have difficulty processing information. A major issue is the lack of self-awareness regarding their condition. Family caregivers understand that these changes are part of the disease, but they find them challenging to manage. An informant describes how his wife screams and says hurtful things to him. She accuses him and uses verbal and physical attacks to express her frustration. This is a new and frightening behavior, which is hard for him to handle, both practically and emotionally.

*Male family caregiver: My wife hits and kicks me and does all sorts of things. It doesn’t hurt me physically*,* but it is unpleasant. I’ve tried to explain it to her*,* but she doesn’t understand.*

When the person with dementia becomes worried, disoriented or experiences anxiety, it negatively affects the health of the informants, causing sleep problems and stress. The informants also highlight the practical challenges that arise when the person wanders away and goes missing from home. Additionally, it is difficult for the family caregivers to have any privacy, even in their own home.

*Male family caregiver: I struggle to have time for myself. My wife accepts that I sometimes go to the computer to pay bills and take care of other tasks. It works for a short while*,* maybe 5–10 min*,* but then she comes and asks what I’m doing.*

The informants experience sadness and a sense of loss as they witness the person’s changes throughout the progression of the disease, which in turn alters their relationship. During time, the person with dementia becomes less proactive and conversations become stilted as their ability to communicate gradually deteriorates. They do not recognize their near ones or are unable to perform basic everyday tasks (using the bathroom, getting dressed, etc.), which can be difficult to cope with emotionally. One informant recounts an incident where her partner developed a high fever and they thought he was going to die, which provoked strong feelings of sadness and desperation. After years of suffering, the informant reflects that it might have been better if the partner had passed away, rather than enduring the slow decline and suffering now going on - thoughts that provoke feelings of guilt. Another informant expresses that he can no longer cope with his wife´s emotional outbursts and blows. He thinks of divorce even though he does not want it. He is grieving the loss of his healthy wife and soon feels like there is no other way out.

#### Isolation and shrinking social networks due to societal stigma and sole caregiving

Caring for a person with dementia takes a lot of time and energy, which often leads to informants being isolated from their social network. Social life and contact with friends and family reduces for both the person with dementia and their family caregivers. Behavioral changes in the person with dementia may trigger negative reactions or avoidance from others, often due to uncertainty about how to manage the situation. Conversely, the person with dementia may struggle to cope with their surroundings. Informants also highlight the challenge of sharing the diagnosis with children and grandchildren, fearing it may cause distancing. The stigma associated with the diagnosis affects the willingness to talk about the disease. Many of those diagnosed with dementia only share their diagnosis with their immediate family and choose not to tell others. This is due to ongoing prejudices around dementia, such as the fear of being seen as less competent or a burden. Some family caregivers believe it is better to proactively inform about the disease to avoid misunderstandings, while others, out of respect for the person with dementia, do not share any information about the disease. This may even prevent family caregivers from joining support groups where they are expected to openly discuss their situation, and out of respect for the wishes of the person with the disease, they may forgo essential support.

*Female family caregiver: There is a support group for family caregivers. I chose not to participate because my husband had a strong need for privacy and didn’t want to talk about it. I felt that I couldn’t talk about my husband. Because everyone spoke about the person*,* they cared for in a way that felt unfamiliar to me.*

Many informants report to sacrifice their own lives and give up leisure activities and social interaction to provide care. Often, the person with dementia refuses to be left alone or attend municipal day care centers.

*Male family caregiver: I have had to give up all my projects. I devote 100% of my time to my wife and I am constantly attentive to her. Sometimes I get a little bit tired of us just sitting here being inseparable*,* just sitting here together all the time. I want to be able to read*,* I have a book that I haven’t been able to make much progress in for several weeks now.*

Persons with dementia are vulnerable and often feel isolated in social situations, which can lead to exclusion and a sense of being disconnected from everyday life. Informants report that the only social opportunity available is the municipal “day care center”. One informant criticizes the low intellectual level of the activities, stating that it discourages his wife from attending. On the other hand, municipal activities designed for people without dementia are often overwhelming as they move too quickly and include too many stimuli for a person with dementia. Family caregivers call for more tailored activities, such as dementia-friendly exercise programs and methods to train the memory methods, to improve well-being.

*Male family caregiver: That didn’t really work either*,* it was too easy for her. There were singing sessions*,* … a sailor comes ashore or white lambs*,* or things like that. That wasn’t her style*,* so she did not want to go there anymore.*

The role of family caregivers is often characterized by mutual dependence. The person with dementia becomes dependent on the family caregiver to speak on their behalf and assist with virtually all daily tasks, while the family caregiver lives according to the needs of the person with dementia. One informant describes this as being co-dependent, like living with someone who has addiction problems. This co-dependence role often forces family caregivers to hide or downplay the illness to protect the person and avoid hurting them. At the same time, they experience an internal dilemma, where their own needs and feelings are sidelined, as their entire life revolves around caring for the person.

*Male family caregiver: I feel like we are co-dependent*,* … I can’t do anything here*,* I try to make sure that my wife is doing well*,* I don’t dare do anything that might upset her. I constantly must check her mood—how is she feeling now? Because it shifts*,* so I must check and adjust myself accordingly. That’s what it means to be co-dependent.*

### Balancing autonomy in care decisions

#### Paradox of full responsibility with limited information

Informants describe the problems that arise when a person with dementia has autonomy over their care, making it difficult for the family caregivers to receive the information and support they need. They describe how challenging it is to establish contact with health care and to accompany the person to medical appointments, as the person with dementia is expected to handle everything themselves. This is further complicated by the fact that the person with dementia struggles to keep track of follow-up appointments, get in contact with the doctor, and follow instructions. Family caregivers are not informed about medical visits or medications if the person with dementia visits the health care center on her own. The informants explain that this autonomy leaves them with no rights, only obligations as they are expected to handle everything from banking to medical care and support.

*Male family caregiver: If my wife says*,* “you can’t go with me to the doctor”*,* then the doctors can’t insist that I be there. Here comes a point when her condition is so severe that she can’t handle the information—no matter what she says. It’s obvious that she can’t make that decision on her own.*

#### Challenges for the person with dementia in recognizing their own care needs

Informants describe the complex and often emotionally charged situations that arise when the need for care and the person’s right to autonomy collide. In cases of moderate dementia, awareness of the illness often diminishes, meaning the person does not understand the level of support they need. The informants note that many older individuals have difficulty accepting help, especially with basic tasks such as personal hygiene. This is particularly challenging for family caregivers when the person lives at home and refuses help from formal caregivers. Another issue arises when someone can no longer live at home due to difficulties managing daily life but refuses to move to a nursing home because autonomy prevents any intervention without consent.

This may lead to people with dementia living in very poor conditions. Informants believe that their voices should be given more weight and that they should have greater influence in decisions regarding care and housing, especially when the person is unable to make rational decisions themselves. One informant describes how the person’s opinion about moving to a nursing home may change from day to day, making it difficult to make long-term decisions, as required by authorities. Finally, this may result in the loss of an available place at the residential care facility.

*Female family caregiver: Family members need to have more of a voice; they (case worker) need to listen to us and then just make the decision. It’s all wrong that people should have to live in complete misery.*

#### Preference to entrust care decisions to professionals

Family caregivers describe the importance of someone external step in, take over responsibility, and deciding that help is necessary *“this is how we will do it now.”* The relationship between the person with a diagnosis and their relative is built on emotions, and the informants do not want to take over or make decisions on behalf of the person.

One informant feels selfish for securing a nursing home place, as if she had abandoned or betrayed the person, experiencing guilt and failure to manage the situation. Often, it is the family caregivers’ declining health that ultimately leads to the decision to apply for a place at a care facility. They feel relieved when receiving help from professionals who take over responsibility and decide. Professionals are also able to recognize signs of deterioration that the family caregivers, with their emotional attachment to the sick person, may not be able to see as objectively.

*Female family caregiver: I found it difficult to sit there and disclose personal information about my mother*,* it felt so wrong. And I feel it’s her job (the case manager). She is a professional*,* she recognizes the signs. For me*,* there is so much emotional baggage and upbringing involved - it’s my mother*,* after all.*

### Dependence on home care and nursing homes that are not adapted to needs

#### Need for home care with dementia expertise, continuity and familiarity

Informants express a need for respite to be able to leave the home, have time for themselves, recover, and take care of their own health. However, they emphasize that home care service should be tailored to their specific situation, as needs and circumstances vary between individuals and at different stages of the illness. Several informants have not used the home care service, while others have tried but were later forced to decline further help because it did not work satisfactorily. In some cases, a lack of continuity made it impossible to continue using home care services. Informants consider it important that home care staff first and foremost build a relationship with the person in need of help, and that, as far as possible, the same staff return regularly. Reactions from the person with dementia vary—some do not mind who comes, while others feel a need for a personal relationship with the person helping them, especially when it comes to intimate tasks like personal hygiene. One informant explain that it was also important for the home care staff to be of the same gender as the person in need of help, as his wife refused to receive help from a male assistant.

*Male family caregiver: One evening a tall*,* dark-haired man stood there and said*,* “I’m here to help your wife wash herself”. It was a disaster*,* she went absolutely mad*,* so we had to send him away.*

The informants also emphasize that home care staff should have basic competence in dementia care, to be able to appropriately interact and communicate with people with dementia. One family caregiver recalls that home care staff tried to force her husband to get dressed properly despite an agreement that he did not need to, resulting in the home care staff fearing the person and waiting outside the home until the informant returned.

#### High threshold for qualifying for nursing home placement

Family caregivers describe the difficulties in securing alternative housing for the person with dementia and the challenges related to the policy of “aging-in-place”. The informants explain that the content in this policy, which aims to keep older people at home as long as possible, can be difficult to assess, as it is not tailored to individual needs, and may be unsustainable for people with dementia.

An informant, a daughter of a person with dementia living alone with support from home care expresses concerns about the person’s dignity and quality of life. The daughter explains that her mother suffers from anxiety, does not eat properly, sleeps poorly, and lacks stimulation. Despite this, she has been denied a place in a nursing home with the reason that the person already receives home care on a regular basis. The informant argues that more flexible options and opportunities for older people to live together are needed, especially for those who are lonely. She believes that her mother´s anxiety could be alleviated if she lived in an environment with constant human presence and continuous care, where proper meals, social interaction, stimulation, and physical activity are provided.

The informants feel that they often have to resort to threats to obtain the necessary care for their relatives. They feel that a lack of individual assessments makes the situation more difficult. There is also a perception that care needs are measured mathematically, e.g., calculations that home care is cheaper and the limited number of available spots, rather than based on human considerations. Additionally, the problem of overcrowded dementia care facilities and the general lack of resources for older people with dementia is highlighted.

*Male family caregiver: There is a mathematical formula*,* but no human assessment of the situation when it comes to getting a place in a nursing home. It’s just numbers. If she needs home care seven times a day*,* she can get a spot*,* but not if she only needs help six times a day. Even if she soils herself or injures herself*,* no*,* there is no place.*

## Discussion

Family caregivers described how caregiving profoundly transformed their lives. While many expressed a strong commitment to taking on this role, they also reported significant negative impacts on their own well-being. Additionally, they highlighted the need for greater and more effective formal support from health care providers, local municipalities, and society.

Family caregivers described how they navigate a challenging paradox: they bear full responsibility for the person with dementia, including managing medical appointments, medications and daily physical and social activities, yet often have limited access to essential information from health care providers, and in some cases, no direct contact with them. In line with a previous study [24], our results show that many family caregivers provide care and support without having the necessary information to fully comprehend the illness, its nature, and how it develops. This situation is further complicated by the cognitive and emotional challenges faced by people with dementia, who may struggle to recognize their own care needs. As a result, family caregivers are often left to make critical decisions on their behalf sometimes against their will. Prioritizing autonomy can sometimes jeopardize the well-being and safety of the person with dementia or their families. Previous research on family caregivers’ experiences aligns with our findings, showing that decision-making, especially in the case of involuntary treatment, is both stressful and overwhelming [25]. Balancing safety and autonomy are difficult, and family caregivers are constantly seeking solutions as dementia symptoms progress. They feel a deep responsibility and face social pressure to ensure the safety of the person with dementia, often fearing blame if something goes wrong [25].

In addition to the constant need for availability, the challenges of managing behavioural changes often trigger mixed emotions, including grief over lost relationships and guilt for experiencing these negative feelings. Previous research has shown that stress from managing behavioral problems, along with a longer duration of caregiving, is strongly associated with negative outcomes for family caregivers. These include exhaustion, stress, and a persistent fear of doing something wrong, fear that may worsen the situation or the disease itself, potentially triggering problematic behaviors instead of alleviating them [10, 11, 14]. Furthermore, informants emphasized the challenges associated with personality changes in the person with dementia, describing how these changes caused significant emotional and social strain. Likewise, prior research has identified several key contributors to caregiver burden, including dementia severity, behavioral symptoms, alterations in personality, and the presence of psychiatric manifestations in the individual with dementia [12].

The societal stigma associated with dementia diagnosis, along with the burden of sole responsibility, often resulted in shrinking social networks and feelings of isolation for family caregivers in this study. Similarly, systematic reviews highlights that many family caregivers often give up their personal interests, reduce time spent with family and friends, and either leave their jobs or cut back on working hours [11, 26]. These sacrifices can lead to a diminished sense of purpose in life, as caregivers prioritize the needs of the person they are supporting over their own well-being. This loss of personal fulfillment may also increase the risk of mental health challenges over time [11]. Many informants were hesitant to seek support from formal care services and preferred to manage everything on their own. This decision was partly based on their belief that it was expected to cope with caregiving independently, both by society and themselves, and partly from dissatisfaction with the quality of available home care services. Caring for a family member with dementia can be perceived as a moral obligation or duty and family caregivers driven by a sense of duty, guilt, or societal expectations are more prone to gradually isolating themselves and experiencing psychological distress [27]. High stress levels among family caregivers are associated with adverse health outcomes for people with dementia, including an increased risk of institutionalization, worsening behavioral and psychological symptoms of dementia (BPSD), incidents of elder abuse, cognitive and emotional decline, poorer quality of life, nutritional problems, and greater health care use and costs [28]. While causality may be bidirectional—where the progression of the care recipient’s condition negatively affects the caregiver—both previous research [28] and our findings highlight the substantial impact of family caregiving, underscoring the urgent need for improved support from health care systems and society.

Our results and other research suggest that there is a discrepancy between the support that family caregivers consider essential and the assistance they actually receive from home care workers [29, 30]. For family caregivers to feel confident in utilizing home care services, continuity of care is essential, along with ensuring that staff are adequately trained and experienced in dementia care. Providing care for people with dementia requires home care workers to strike a balance between respecting the person’s autonomy and privacy while ensuring their safety and dignity [24, 31, 32]. Unless these factors are addressed it is challenging for family caregivers to entrust the care of their spouse or parent to home care services. Quality dementia care relies heavily on having a deep understanding of the individual and effectively managing challenging behaviors with empathy and person-centered strategies that address the underlying needs and feelings behind these behaviors [31]. In this study, the informants emphasized that competent home care staff are those who can communicate according to the person’s abilities, maintain continuity so that the staff know the individual and their family, and understand how to appropriately support and interact with a person with dementia.

Our study and earlier research underscore the importance of person-centered support tailored to the unique requirements of both the family caregiver and the person with dementia. This highlights the importance of person- and family-centered dementia care, which not only focuses on the individual’s needs, values, goals, and preferences, but also considers the family’s situation and involvement. Family-centered care builds on person-centered care by recognizing the vital role of family members and encouraging their respectful and meaningful participation in care planning and decision-making [33]. A standardized, one-size-fits-all approach fails to adequately address the diverse and complex challenges faced by family caregivers [34]. However, implementing person- and family-centered support requires a well-trained workforce that specializes in dementia care [17, 33, 35, 36]. In Sweden, the majority of home care workers have minimal, or no formal education witch significantly impacts their ability to meet the complex needs of individuals with dementia and their family [37, 38]. Based on this study and previous research, suggestions for interventions to enhance dementia care and support family caregivers include strengthening policies on educational requirements by mandating specialized training programs for home care workers to enhance their competence in dementia care [17, 35, 36]. It is also important to implement person- and family-centered care approaches that address the needs of both persons with dementia and their family caregivers [29, 33]. Person- and family-centered dementia care focuses on three core components; (1) *Respect and dignity-*Health care providers actively listen to and value the perspectives and choices of both the person with dementia and their families. The knowledge, values, beliefs, and cultural backgrounds of persons and families are integrated into care planning and delivery. *(2) Information sharing-* Health care professionals communicate openly and transparently, providing the person with dementia and families with complete, unbiased, and relevant information. This enables them to participate effectively in their care and make informed decisions in a timely manner. (3) *Participation and Collaboration-* The person with dementia and their families are encouraged and supported to engage in care and decision-making according to their desired level of involvement [33]. These approaches should be developed collaboratively with family caregivers, the person with dementia, social services, and health care professionals, recognizing the home as central to the family’s identity. Furthermore, it is important to raise awareness among health care and social care professionals to better recognize and address the emotional, physical, and mental health needs of family caregivers [39, 40].

## Strengths and limitations

This study has some methodological limitations. First, all informants were recruited through the Swedish Dementia Association channels and contacts, which may have led to the inclusion only of individuals who are actively engaged on the topic. However, the inclusion criteria aligned with recruiting informants who were open to discussing their experiences. Second, the quality of the data may be affected because the original aim of the study was not specifically on the everyday lives of family caregivers to people with dementia. This might have limited the ability to explore the topic fully, which could have impacted on the depth of the findings. Although we included both spouses and adult children in the sample, which enabled us to capture a variety of perspectives, we acknowledge that their experiences of supporting a person with dementia may differ significantly. Spouses often share a long-term partnership and daily life with the person, while adult children may have more limited physical proximity but still experience emotional and practical burdens [15]. This variation may have influenced the findings, and future studies could consider examining these groups separately to further explore relationship-specific support needs. Another limitation of the study is that we did not collect detailed demographic information regarding the duration of caregiving or the stage of dementia of the care recipients. As the primary focus was on family caregivers’ subjective experiences, such contextual factors were not systematically recorded. We acknowledge, however, that these variables may influence caregiving experiences and could have provided additional nuance to the analysis. Future studies may benefit from including this type of information to explore how the progression of dementia and length of caregiving impact family caregivers’ needs and perceptions.

Furthermore, as our informants were drawn from a specific region that is predominantly affluent and ethnically homogeneous, the applicability of the findings to other settings may be limited. In addition, while our sample size was guided by the concept of information power [22], we acknowledge that the relatively small and regionally specific sample may limit the diversity of perspectives. This may affect the transferability of the findings to other contexts. By transparently reporting our methodological processes, we aim to enable readers to assess the transferability of our findings to other contexts.

The analysis benefited from the involvement of multiple researchers with diverse expertise in the medical, nursing, epidemiological and sociological fields, as well as two coders who compared and discussed similarities and differences during the coding process to develop the categories. To ensure that all relevant perspectives were considered, the research team also presented the interview results to the reference group at the Swedish Dementia Association (www.demensforbundet.se).

## Conclusions

Caring for a family member with dementia transforms relationships, as family caregiver takes on responsibility for daily life, including social and health care needs. Balancing safety and well-being with emotional and physical health for the person with dementia, while navigating limited support, remains a constant challenge. Professional support should be both person- and family- centered, and developed in partnership with family caregivers, recognizing the home as central to the family’s identity. Enhanced support from health care and social services, along with a skilled and consistent home care workforce, is crucial for the well-being of both the person with dementia and the caregiver. To support wider use of person- and family-centered dementia care, policies should ensure key principles are upheld, including adequate resources, coordinated care, trained interdisciplinary teams centered on the person with dementia and family, and services that reflect what matters most to them. However, further conceptual development is needed to better understand what constitutes best practices in person- and family-centered dementia care.

## Data Availability

No datasets were generated or analysed during the current study.
